# Investigation of LuxS-mediated quorum sensing in *Klebsiella pneumoniae*

**DOI:** 10.1099/jmm.0.001148

**Published:** 2020-01-28

**Authors:** Lijiang Chen, Jonathan J. Wilksch, Haiyang Liu, Xiaoxiao Zhang, Von V. L. Torres, Wenzi Bi, Eric Mandela, Jianming Cao, Jiahui Li, Trevor Lithgow, Tieli Zhou

**Affiliations:** ^1^​ Department of Clinical Laboratory, The First Affiliated Hospital of Wenzhou Medical University, Wenzhou, Zhejiang Province, PR China; ^2^​ Infection and Immunity Program, Biomedicine Discovery Institute and Department of Microbiology, Monash University, Clayton, Victoria, Australia; ^3^​ Department of Infectious Diseases, Sir Run Run Shaw Hospital, College of Medicine, Zhejiang University Hangzhou, China; Key Laboratory of Microbial Technology and Bioinformatics of Zhejiang Province, Hangzhou, PR China; ^4^​ School of Laboratory Medicine and Life Science, Wenzhou Medical University, Wenzhou, Zhejiang Province, PR China

**Keywords:** quorum sensing, *luxS*, *Klebsiella pneumoniae*, biofilm

## Abstract

**Introduction:**

Autoinducer-2 (AI-2) quorum sensing is a bacterial communication system that responds to cell density. The system requires *luxS* activity to produce AI-2, which can regulate gene expression and processes such as biofilm formation.

**Aim:**

To investigate the role of *luxS* in biofilm formation and gene expression in the nosocomial pathogen *Klebsiella pneumoniae *.

**Methodology:**

A *ΔluxS* gene deletion was made in *K. pneumoniae* KP563, an extensively drug-resistant isolate. AI-2 production was assessed in wild-type and *ΔluxS* strains grown in media supplemented with different carbohydrates. Potential roles of *luxS* in biofilm formation were investigated using a microtiter plate biofilm assay and scanning electron microscopy. Quantitative RT-PCR evaluated the expression of lipopolysaccharide (*wzm* and *wbbM*), polysaccharide (*pgaA*), and type 3 fimbriae (*mrkA*) synthesis genes in wild-type and *ΔluxS* mutant biofilm extracts.

**Results:**

AI-2 production was dependent on the presence of *luxS*. AI-2 accumulation was highest during early stationary phase in media supplemented with glucose, sucrose or glycerol. Changes in biofilm architecture were observed in the *ΔluxS* mutant, with less surface coverage and reduced macrocolony formation; however, no differences in biofilm formation between the wild-type and *ΔluxS* mutant using a microtiter plate assay were observed. In *ΔluxS* mutant biofilm extracts, the expression of *wzm* was down-regulated, and the expression of *pgaA*, which encodes a porin for poly-β−1,6-N-acetyl-d-glucosamine (PNAG) polysaccharide secretion, was upregulated.

**Conclusion:**

Relationships among AI-2-mediated quorum sensing, biofilm formation and gene expression of outer-membrane components were identified in *K. pneumoniae *. These inter-connected processes could be important for bacterial group behaviour and persistence.

## Introduction

Quorum sensing (QS) is a cell-to-cell communication system that allows bacteria to regulate biological functions in response to changes in population density, thus acting as a mechanism for environmental adaptation [1, 2]. The system is controlled by the production, secretion and detection of extracellular signalling molecules called autoinducers. Two main classes of autoinducers are defined by two systems, termed Type I QS and Type II QS. In Type I QS, the autoinducer-1 molecules are *N*-acyl homoserine lactone (AHL) derivatives. In Type II QS, the signalling molecules are known as autoinducer-2 (AI-2) [3, 4]. Unlike Type I QS, which is a highly specific system used for intraspecies communication, Type II QS is believed to function for interspecies communication, allowing bacteria to respond not only to their own AI-2, but to the AI-2 produced by other species.

AI-2 is produced by the enzyme LuxS and converts S-ribosylhomocysteine (SRH) to 4,5-dihydroxy-2,3-pentanedione (DPD) [5]. The DPD form is unstable and undergoes spontaneous cyclization to form a furanosyl borate diester (the AI-2 molecule). In *Escherichia coli *, AI-2 is exported via the transmembrane protein TqsA and imported by the ABC transporter LsrACDB [6, 7]. Once internalised into the cytoplasm, AI-2 is phosphorylated by LsrK, and phospho-AI-2 acts to inhibit LsrR repression of the *lsr* operon, thereby leading to increased AI-2 uptake. As the bacterial cell density increases, and once a critical threshold level of extracellular AI-2 is reached for detection by cognate receptors, a signal transduction cascade is triggered. This signalling results in population-wide expression of target genes and alterations in bacterial physiology relating to virulence, protein secretion, extracellular polysaccharide production, iron acquisition, motility and biofilm formation [4, 8].

The Gram-negative bacterium *Klebsiella pneumoniae* has emerged as a multidrug-resistant pathogen that has spread globally and is acknowledged as a cause of invasive blood-borne infections, as well as pneumonia and urinary tract infections, particularly in healthcare settings [9, 10]. *K. pneumoniae* characteristically produces a thick and often mucoid polysaccharide capsule and an assortment of adherence factors, which assist bacteria to persist in diverse environments, particularly via their attachment to surfaces within biofilm communities [11, 12]. Biofilms provide conditions that physically protect cells from hostile environmental factors, antimicrobials or components of the immune system, and are the cause of many chronic infections, particularly those associated with indwelling medical devices [13–15]. These sessile communities, where bacteria can reside in close proximity with each other, also creates increased opportunities for chemical signalling and gene transfer to occur between bacterial cells of the same or different species [16, 17]. This is an especially important genetic mechanism that is largely responsible for the continually increasing incidence of antibiotic-resistant phenotypes and related infections [18]. Recent efforts to develop alternative strategies to combat bacterial infections have led to the identification of novel compounds that target bacterial processes, including quorum sensing and biofilm formation [19–21]. For instance, a ‘quorum quenching’ enzyme that inactivates AI-2 molecules has been discovered that inhibits *K. pneumoniae* biofilm formation [22].

A functional Type II QS system was previously identified in *K. pneumoniae *, where *luxS* was shown to be critical for AI-2 synthesis [5, 23], and mutations in quorum sensing-related genes induced changes in biofilm formation and LPS synthesis [5, 24]. However, other biological processes and genetic targets regulated by QS in *K. pneumoniae* have yet to be identified. This study aimed to assess whether a *ΔluxS* mutant of an extensively drug-resistant *K. pneumoniae* clinical isolate demonstrated changes in biofilm formation and gene expression. The study also examined how environmental cues, in the form of various carbon sources, regulate the production of AI-2 by *K. pneumoniae *.

## Methods

### Bacterial strains, plasmids and growth conditions

The bacterial strains and plasmids used in this study are listed in Table 1. *K. pneumoniae* strain KP563 is an extensively drug-resistant clinical strain isolated in 2006 from the First Affiliated Hospital of Wenzhou Medical University, Wenzhou, China [25]. *K. pneumoniae* and *E. coli* strains were grown in Luria-Bertani (LB) media or LB supplemented with 1 % (w/v) glucose, 1 % (w/v) sucrose or 1 % (w/v) glycerol at 37 °C with shaking or as static cultures, as required. *Vibrio harveyi* BB170 (*luxN*::tn*5*Kan^R^, AI-1^-^, AI-2^+^) was cultivated in autoinducer bioassay (AB) medium at 30 °C with shaking [26]. When appropriate, antibiotics were added at the following concentrations: ampicillin (Amp; 100 μg ml^−1^), kanamycin (Kan; 30 μg ml^−1^), chloramphenicol (Chl; 60 μg ml^−1^) or tetracycline (Tet; 12.5 μg ml^−1^).

**Table 1.** Bacterial strains and plasmids used in this study*

Strain/plasmid	Relevant genotype and properties	Source/reference
**Strains**		
*** Klebsiella pneumoniae ***		
KP563 WT	Wild-type, clinical isolate; Amp^R^ Cfz^R^ Caz^R^ CRO^R^ Azt^R^ Gm^R^	Laboratory stock
KP563 Δ*luxS*	KP563 *luxS*::*km deletion mutant*.	This study
*** Vibrio harveyi ***		
BB170	*luxN*::Tn5 (S-1^-^,S-2^-^), sensor 1^-^, sensor 2^-^, reporter strain	[31]
**Plasmids**		
pluxS	pACYC184 containing the KP563 luxS gene inserted into the Tet^R^ gene; Chl^R^	This study
pGEM-luxS:kan	pGEM-T Easy containing *luxS* flanking sequences and Km^R^ cassette used for gene gorging; Amp^R^ Km^R^	This study
pGEM-T Easy	High-copy-number, cloning vector for PCR products; Amp^R^	Promega
pACYC184	Medium-copy-number, cloning vector, p15A ori; Tet^R^ Chl^R^	[30]
pKD4	Source of FRT-flanked Kan^R^ cassette; Amp^R^ Km^R^	[28]
pACBSR	Mutagenesis plasmid used for gene gorging. Ara promoter control, I-SceI and λ Red recombinase; Chl^R^	[27]

*Abbreviations: Amp, ampicillin; Cfz, cefazolin; Caz, ceftazidime; CRO, ceftriaxone; Azt, aztreonam; Gm, gentamicin; Km, kanamycin; Tet, tetracycline; Chl, chloramphenicol; Nal, nalidixic acid.

### Construction of *K. pneumoniae ΔluxS* mutant

A *K. pneumoniae* KP563 *luxS-*deficient (Δ*luxS*) mutant was constructed by allelic replacement with a kanamycin resistance-encoding gene (*Km*) following the ‘gene gorging’ method, as described previously [27]. The required primers were designed using the reference *K. pneumoniae* MGH78578 genome and listed in Table 2. The ‘donor plasmid’ containing the desired mutation was constructed as follows. A 1478 bp *Km* cassette with flanking fragment length polymorphism (FLP) recombinase target (FRT) sites was amplified from pKD4 using primers KanF and KanR [28]. The 492 and 699 bp fragments flanking the upstream and downstream *luxS* gene sequence, respectively, were amplified from *K. pneumoniae* KP563 genomic DNA. Overlapping extension PCR [29] was used to connect the three fragments to yield a ~2.7 kb ISce-I-flanked product, which was cloned into pGEM-T Easy (Promega) to create the donor plasmid. The construct was confirmed by DNA sequencing.

**Table 2.** Oligonucleotide primers used in this study*

Primer name	Sequence (5′−3′)
luxS(SceI)-F	TAGGGATAACAGGGTAATACAGTATCCACGGTGGCCAG
luxS(Kan)-R	**GAAGCAGCTCCAGCCTACACA**TTCGGGTATGGTCGACTGTG
luxS(Kan)-F	**CTAAGGAGGATATTCATATG**TCATATCATTGAGCGCGATG
luxS(SceI)-R	TAGGGATAACAGGGTAATGGTTTAGGGTTCGCTCGCTC
Kan-F	TGTGTAGGCTGGAGCTGCTTC
Kan-R	CATATGAATATCCTCCTTAG
Kan-R2	GAAGCAGCTCCAGCCTACACA
luxS(BspHI)-F	TCATGATGAGTATATCGAAGTGCGTTCG
luxS(BamHI)-R	GGATCCAGTCATGTTGATGCCAGTCTTCC
wzm-F	TGCCAGTTCGGCCACTAAC
wzm-R	GACAACAATAACCGGGATGG
wbbM-F	ATGCGGGTGAGAACAAACCA
wbbM-R	AGCCGCTAACGACATCTGAC
pgaA-F	GCAGACGCTCTCCTATGTC
pgaA-R	GCCGAGAGCAGGGGAATC
mrkA-F	AGCGATGCGAACGTTTACCTGTCTC
mrkA-R	CGTCATCCTGTTTAGTGCCATCAGC
rpoB-F	AAGGCGAATCCAGCTTGTTCAGC
rpoB-R	TGACGTTGCATGTTCGCACCCATCA

*Recognition sequences for restriction enzymes are underlined. Kanamycin resistance-encoding gene-specific sequences are in boldface. F, forward (5′) primer. R, reverse (3′) primer.

The mutagenesis was conducted by electroporating the donor plasmid and pACBSR (which contains I-SceI endonuclease and lambda Red recombinase genes under inducible control by l-arabinose) into competent *K. pneumoniae* cells [27]. Transformants were selected on LB agar plates containing kanamycin and chloramphenicol. One transformant was inoculated into 1 ml LB supplemented with 0.2 % (w/v) l-arabinose and chloramphenicol and cultured at 37 °C for 16 h. Cell dilutions were grown on LB agar containing kanamycin, and mutants were confirmed by colony PCR using luxS(BspHI)F/luxS(BamHI)R and luxS(BspHI)F/KanR2 primers. The loss of pACBSR was induced by 0.2 % l-arabinose without selection.

### Creation of complementation constructs

The *K. pneumoniae* KP563 *luxS* gene and native promoter region was amplified by PCR using luxS(BspHI)F and luxS(BamHI)R primers and cloned into pACYC184 [30] via the unique BamHI/BspHI restriction sites within the tetracycline resistance-encoding gene. Complementation constructs were maintained in cells with chloramphenicol resistance selection. The empty vector (pACYC184) was introduced into *K. pneumoniae* wild-type and Δ*luxS* mutant strains as controls.

### AI-2 production assay

Detection of AI-2 in the *K. pneumoniae* culture supernatants was assessed using the reporter strain *V. harveyi* BB170 [31], as described previously [26]. Briefly, *K. pneumoniae* strains were grown in LB or LB containing 1 % glucose, 1 % sucrose or 1 % glycerol, with shaking at 37 °C. Cell-free supernatant (CFS) samples were prepared every 2 h by centrifugation (10 min at 8000 ***g***) of 1 ml aliquots, followed by passing through 0.2 µm membrane filters. CFS samples were stored at −20 °C until assayed. A 180 µl aliquot of an overnight culture of *V. harveyi* BB170 was mixed with 20 µl CFS sample and diluted 1 : 5000 in AB medium. Samples were then transferred to black 96-well microtiter plates with flat transparent bottom (Corning) and incubated at 30 °C for 3 h with shaking. AI-2 levels were measured in an Infinite M200 plate reader (Tecan) at OD_490_ and data were represented as the fold change relative to the negative control (AB medium replaced CFS).

### Growth curve measurement

Stationary *K. pneumoniae* cultures were diluted 1 : 1000 in LB, or LB supplemented with 1 % glucose, 1 % sucrose, or 1 % glycerol, and incubated at 37 °C with shaking. Growth was measured every 2 h at OD_600_ using an Infinite M200 plate reader. Experiments were conducted in triplicate.

### Biofilm formation assay

Biofilm formation assays were performed as previously described with minor modifications [32]. Strains were initially grown in LB overnight at 37 °C before diluting 1 : 100 in LB, or LB supplemented with 1 % glucose, 1 % sucrose, or 1 % glycerol, and aliquoting 100 µl into 96-well, flat bottom, non-tissue culture treated polystyrene plates (Corning). Wells containing media alone were used as negative controls. Following incubation for 24 h at 37 °C, planktonic cells were removed and the wells were washed twice with dH_2_O. Biofilms were stained with 150 µl 0.1 % (w/v) crystal violet for 15 min and wells were rinsed twice with dH_2_O. Stained biofilms were solubilised with 95 % ethanol and quantified by measuring the OD_600_ using an Infinite M200 plate reader.

### Scanning electron microscopy (SEM)

Stainless steel pieces were incubated with *K. pneumoniae* in LB for 24 h at 37 °C, as described elsewhere [33]. Samples were fixed in 2.5 % glutaraldehyde for 2 h and then exposed to increasing concentrations of ethanol (50 %, 70 %, 80 %, 90 %, 100 %) for 10 min each. Biofilms were then dried with hexamethyldisilazane and coated with gold. The biofilms on stainless steel were examined with an SEM (Hitachi S-3000N, Japan). Images were captured at ×2000 magnification.

### Quantitative reverse transcription (RT)-PCR

The expression levels of *mrkA*, *wzm*, *wbbM* and *pgaA* genes in *K. pneumoniae* KP563 wild-type and Δ*luxS* were determined from biofilm-grown cells using quantitative RT-PCR (qRT-PCR). Briefly, bacteria were grown in RPMI 1640 at 37 °C in 6-well microtiter plates and, after 8- or 24 h, wells were gently washed and biofilm cells were removed from the well surface using a cell scraper and resuspended in saline solution. Total RNA was extracted from *K. pneumoniae* using an RNeasy Mini Kit (Qiagen) and cDNA was synthesised using the RevertAid First Strand cDNA Synthesis Kit (Thermo Scientific) according to the manufacturers’ instructions. qRT-PCR was performed with an Applied Biosystems 7500 RT-PCR System using a SYBR Green RT-PCR Kit (Qiagen) and the primer pairs listed in Table 2. The relative expression levels of tested genes were normalised to the expression of the *rpoB* housekeeping gene. Each sample was run in triplicate and the means of Ct values were obtained for analysis. The relative gene expression was represented as fold change between the *K. pneumoniae* KP563 wild-type and Δ*luxS* mutant strains. Data were analysed using the 2^-ΔΔCt^ method.

### Statistical analysis

The Student’s *t*-test was used to analyse the difference in biofilm formation ability and gene expression levels. *P* values of<0.05 were considered statistically significant. Statistical analysis was performed with GraphPad Prism 7.01 software.

## Results

### Construction of a *K. pneumoniae* Δ*luxS* mutant

**Fig. 1.**
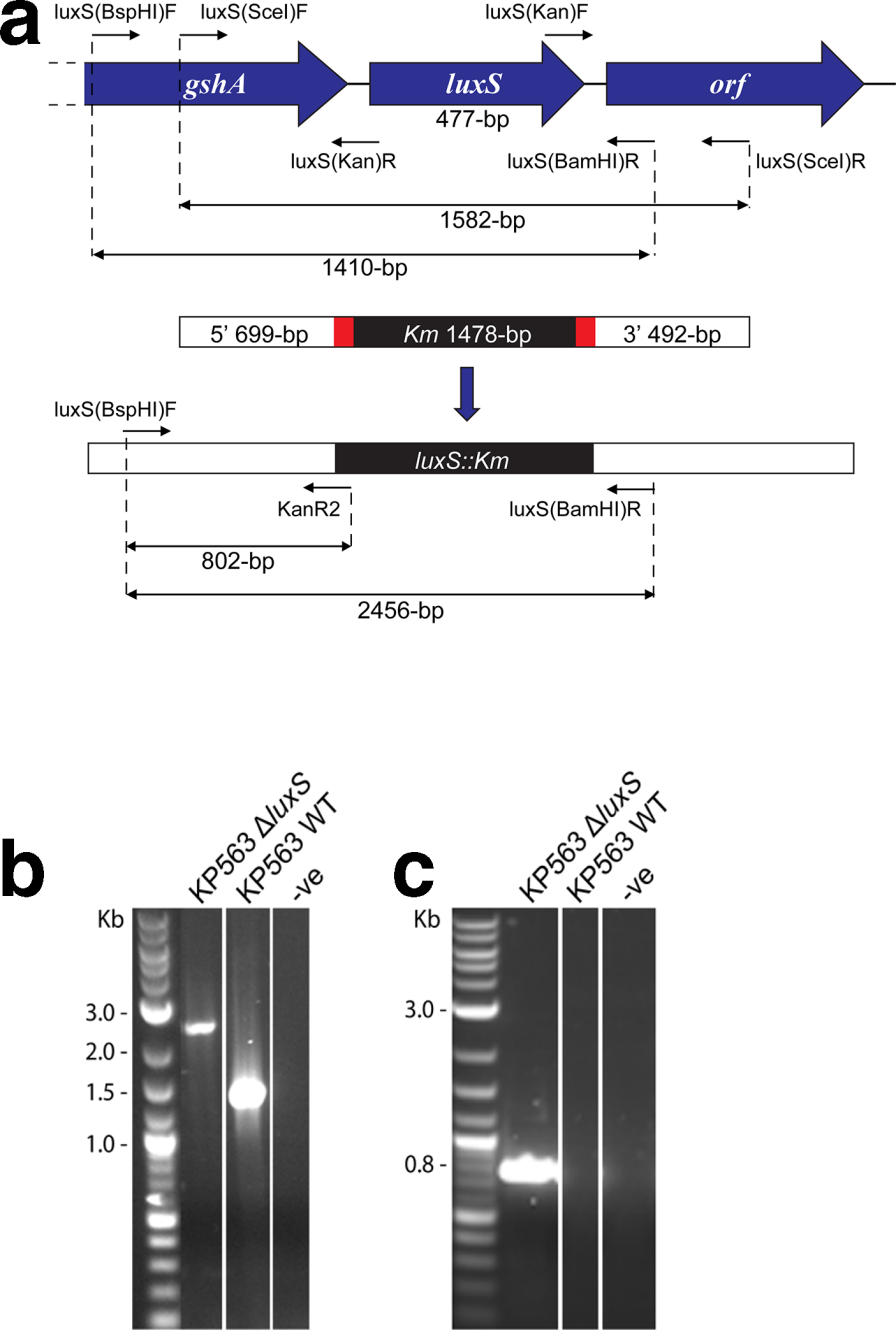
(a) Generation of a *K. pneumoniae* KP563 Δ*luxS* mutant. A genetic map is shown of *luxS* and flanking genes (*gshA* and an unannotated ORF) in KP563. Short arrows represent approximate locations and directions of primers used. A Δ*luxS* knockout strain was created via allelic exchange with a DNA fragment containing the kanamycin resistance cassette (*Km*) flanked by upstream (5′ 699 bp) and downstream (3′ 492 bp) sequences from *luxS*, as detailed in the Materials and Methods. The regions in red represent 20 bp complementary sequences between the flanking and *Km* sequences, required for overlapping PCR. Expected amplicon lengths are shown. (b and c) Confirmation of KP563 Δ*luxS* mutant by PCR. Colony PCR was performed on KP563 wild-type (WT) and Δ*luxS* mutant using (b) Primer pair 1 luxS(BspHI)F/luxS(BamHI)R, and (c) Primer pair 2 luxS(BspHI)F/KanR2. Primer pair 1 produced an expected ~1 kb product size difference. Primer pair 2 produced an expected ~0.8 kb product for the Δ*luxS* mutant only. Negative (-ve) is the no-template control.

To analyse the function of *luxS*-dependent signalling in *K. pneumoniae* KP563, we first constructed an isogenic Δ*luxS* mutant in this strain by replacing the *luxS* gene with a kanamycin resistance cassette via allelic exchange (Fig. 1a). The mutant strain was verified by PCR (Fig. 1b and c) and sequencing.

### Effect of carbon source on AI-2 production by *K. pneumoniae*

A bioluminescence assay was used to monitor the kinetics of AI-2 production in *K. pneumoniae *. Comparisons were made for *K. pneumoniae* KP563 wild-type, Δ*luxS* mutant and the Δ*luxS* mutant complemented with pACYC184 carrying the KP563 *luxS* gene (pluxS). At various time-points during growth, the cell-free supernatant was collected and tested for luminescence activation in a *V. harveyi* BB170 reporter strain, which is known to respond to AI-2 [31, 34]. The AI-2 levels reported in the assay reflect the net production and degradation rates of the autoinducer.

**Fig. 2.**
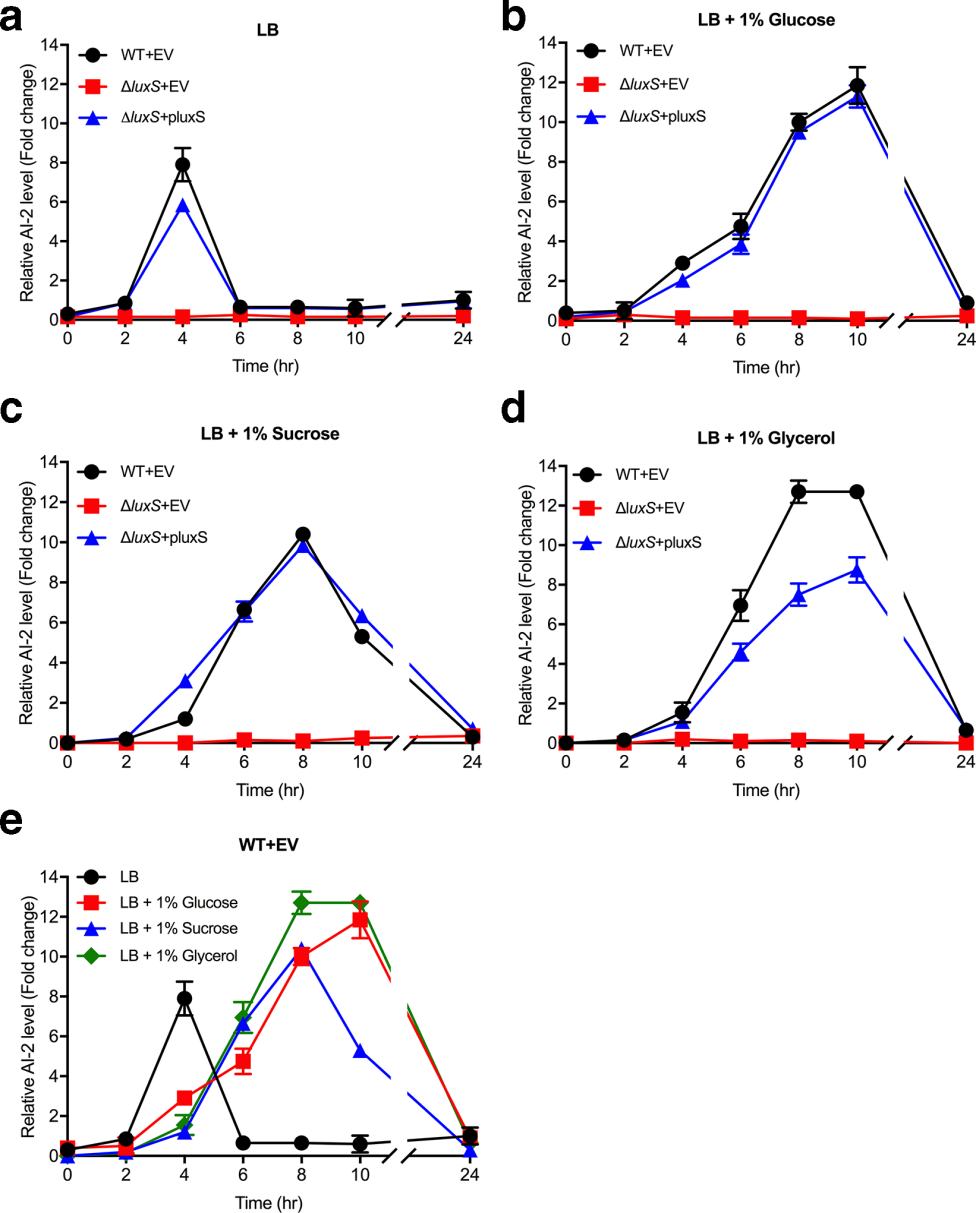
AI-2 production by *K. pneumoniae* KP563 wild-type and Δ*luxS* mutant strains in different media. Strains were grown in (a) LB and LB containing (b) 1 % glucose, (c) 1 % sucrose and (d) 1 % glycerol. (e) Represents wild-type *K. pneumoniae* KP563 grown in the above media conditions. *K. pneumoniae* cultures were incubated at 37 °C with shaking. Supernatants were collected at the indicated time intervals and mixed with the *V. harveyi* BB170 reporter strain to detect AI-2 expression. EV=empty vector (pACYC184). Data are the means of three experimental replicates and are represented as fold change relative to a negative control. Error bars represent 95 % confidence intervals.

**Fig. 3.**
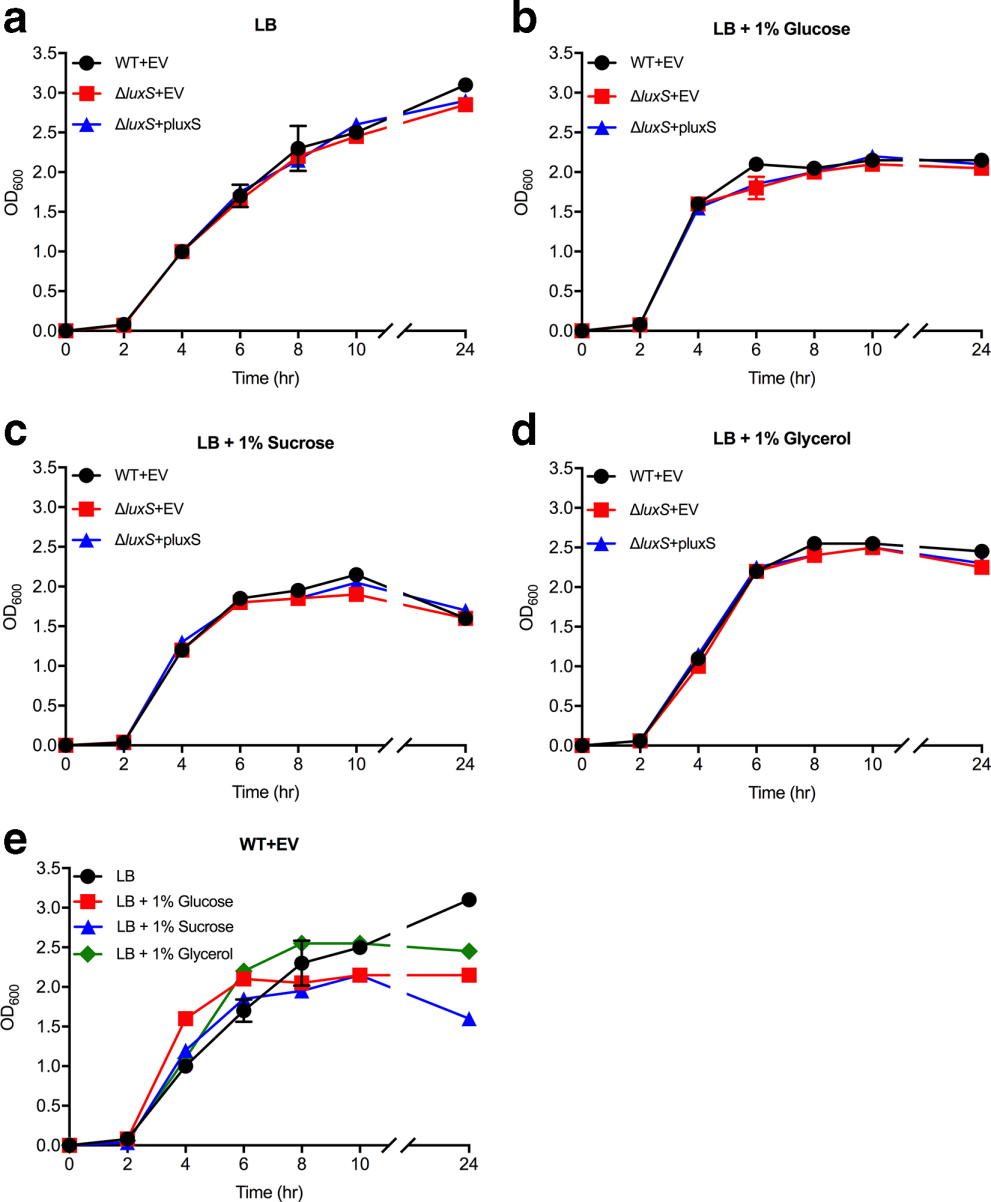
Growth curves of *K. pneumoniae* KP563 wild-type and Δ*luxS* mutant strains in different media. Strains were grown in (a) LB and LB containing (b) 1 % glucose, (c) 1 % sucrose and (d) 1 % glycerol. (e) Represents growth of wild-type *K. pneumoniae* KP563 in the above media conditions. *K. pneumoniae* cultures were incubated at 37 °C with shaking and absorbance readings (OD_600_) were taken at the indicated time intervals. EV=empty vector (pACYC184). Data are the means of three experimental replicates. Error bars represent 95 % confidence intervals.

As shown in Figs 2a, AI-2 production by *K. pneumoniae* cultured in LB media was detected between 2–6 h growth and reached peak production at the mid-log growth phase (~4 h). In contrast, the Δ*luxS* mutant failed to produce detectable levels of AI-2 over 24 h. Complementation of the Δ*luxS* mutant with the pluxS plasmid restored AI-2 production to near wild-type levels. To identify media conditions that promoted AI-2 production in *K. pneumoniae* KP563, 1 % glucose, sucrose or glycerol were supplemented to LB media. The exogenous carbon sources caused significant changes in the rates of AI-2 accumulation by *K. pneumoniae* (Fig. 2b–e). LB media containing any of these carbohydrates also resulted in *K. pneumoniae* reaching stationary growth phase at an earlier time (~6 h) compared to LB without supplementation (>10 h) (Fig. 3). In the presence of the supplemented sugars, AI-2 levels gradually accumulated after 2 h and reached a maximum level during early stationary growth phase (~8–10 h) before falling to barely detectable levels by 24 h. As observed previously, AI-2 production was not detected in the Δ*luxS* mutant, however partial or complete AI-2 production was observed upon pluxS complementation. There were no observable differences in growth rate between the wild-type, Δ*luxS* mutant and complemented Δ*luxS*+pluxS mutant in all conditions tested (Fig. 3).

### Investigation of the contribution of LuxS to biofilm formation

**Fig. 4.**
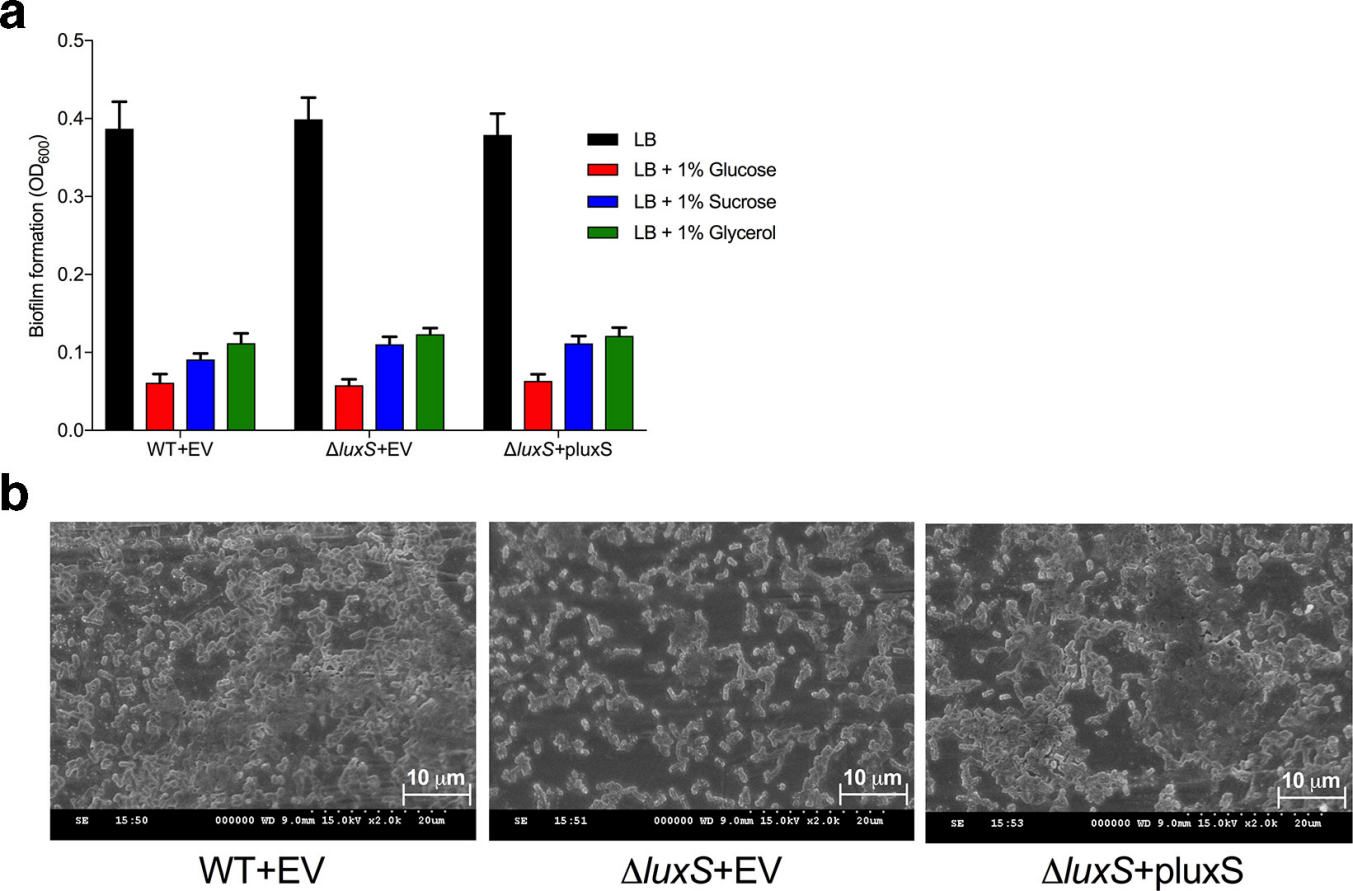
Influence of different carbon sources on biofilm formation by *K. pneumoniae* KP563. (a) *K. pneumoniae* strains were grown in LB or LB supplemented with carbon sources within wells of 96-well non-treated polystyrene microtiter plates for 24 h at 37 °C. Biofilms were stained with crystal violet and quantified by measuring the OD_600_. Data are the means of two experimental replicates (total number of wells=10). Error bars represent 95 % confidence intervals. (b) Representative SEM images of *K. pneumoniae* attached to stainless steel pieces following incubation in LB for 24 h at 37 °C. Images are at ×2000 magnification. EV=empty vector (pACYC184).

To explore what influence a Δ*luxS* mutation had on the biofilm-forming ability of *K. pneumoniae* KP563, a crystal violet stain-based biofilm assay using 96-well polystyrene plates as the substrate was employed. No difference was observed between the wild-type and Δ*luxS* mutant strains when cultured in LB for 24 h (Fig. 4a). The addition of different carbon sources to the growth media, whilst significantly reducing the amount of biofilm formation for all strains tested, did not cause a difference in biofilm formation between the wild-type and Δ*luxS* mutant.

Scanning electron microscopy (SEM) was then used to examine more closely the structure of the biofilm architecture. The imaging revealed that the biofilms of *K. pneumoniae* KP563 wild-type and complemented Δ*luxS*+pluxS mutant strains appeared dense and aggregated with evidence of large regions of macrocolony formation. In contrast, the Δ*luxS* mutant cells were more sparsely localised within the biofilm mass (Fig. 4b).

### LuxS-mediated alteration in biofilm-related gene expression

**Fig. 5.**
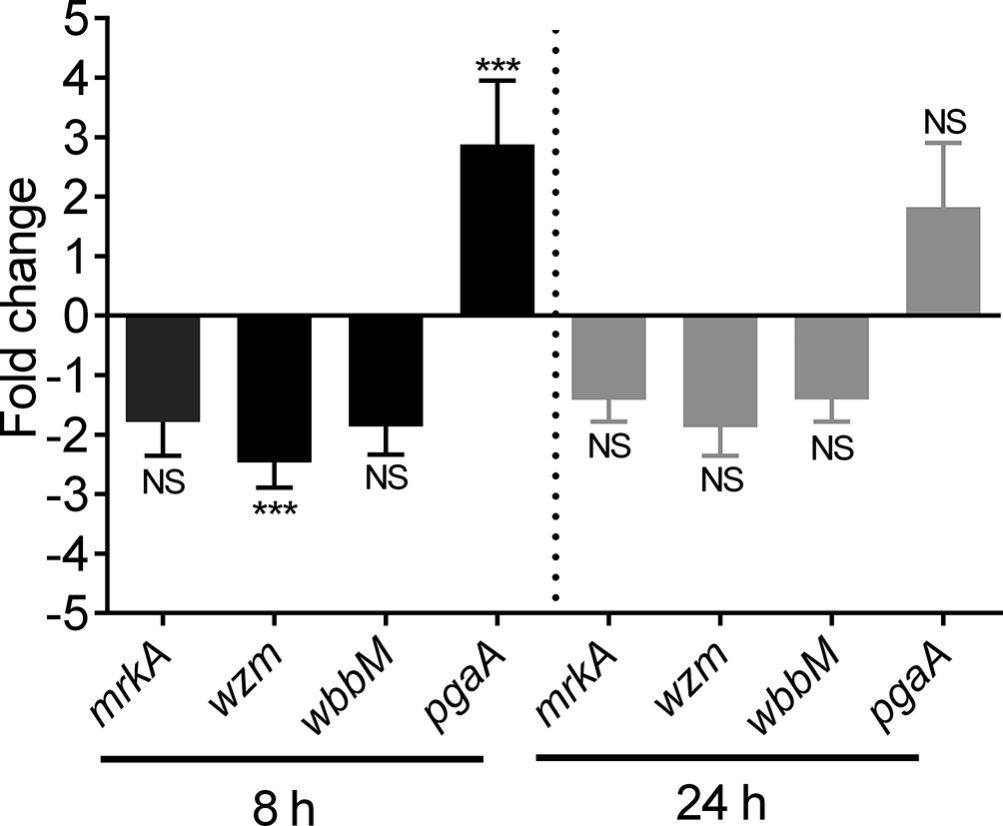
Analysis of gene expression levels in 8- and 24 h biofilm extracts of *K. pneumoniae* wild-type and *ΔluxS* mutant. Data represent the fold change in the relative gene expression level in the *ΔluxS* mutant relative to the wild-type strain. Target gene expression was normalised to *rpoB*. Data are the means of three experimental replicates. *P* values were calculated using Student's *t*-test (***P<0.001; NS: not significant, *P*>0.05). Error bars represent 95 % confidence intervals.

Given the differences observed in macrocolony formation observed above, we quantified the expression of four genes in the wild-type and *ΔluxS* mutant that are involved in the production of prominent outer-membrane structures. These included genes required for lipopolysaccharide (LPS) biosynthesis (*wzm* and *wbbM*), poly-β−1,6-*N*-acetyl-d-glucosamine (PNAG) polysaccharide secretion (*pgaA*) and type 3 fimbriae biosynthesis (*mrkA*). Quantitative RT-PCR was performed on RNA extracted from *K. pneumoniae* wild-type and *ΔluxS* mutant biofilm extracts following 8- and 24 h growth, and the relative gene expression was determined. Compared to their expression in the wild-type strain, there were no significant differences in the expression of *mrkA* and *wbbM* genes in the *ΔluxS* mutant after 8- or 24 h biofilm growth (Fig. 5). However, a significant decrease in the expression of *wzm* (2.7-fold) and an increase in the expression of *pgaA* (2.9-fold) in the *ΔluxS* mutant compared to the wild-type strain were observed after 8 h biofilm growth.

## Discussion

### Influence of growth conditions on AI-2 production

Using a *V. harveyi* reporter system that responds to AI-2 molecules, we showed that *K. pneumoniae* KP563 grown in LB without supplemented carbon produced maximum AI-2 levels during the mid-exponential phase. In addition, AI-2 production was abolished in the Δ*luxS* mutant when tested in all media conditions, which supports other studies suggesting that Type II QS is *luxS*-dependent in *K. pneumoniae* [5, 35]. We also showed that the *K. pneumoniae* KP563 *luxS* gene under the control of its native promoter could trans-complement the Δ*luxS* mutation. These results are also consistent with other studies showing that maximum AI-2 production is detected during the exponential phase, which is also the period of greatest *luxS* gene expression levels [5, 23, 35]. However, AI-2 levels returned quickly to basal levels in the late-exponential phase, before cell density had reached its maximum. This is in contrast with other *K. pneumoniae* strains that show AI-2 accumulation into the early stationary phase when the bacterial density reaches its highest [5, 23]. In *E. coli* and *S. enterica* serovar Typhimurium, only low levels of AI-2 were detected in the culture supernatant when grown in LB in the absence of glucose, due primarily to the rapid internalisation of AI-2 into cells by the Lsr transporter [7, 36]. Thus, in the absence of an exogenous carbon source, extracellular AI-2 exists only transiently.

It has been shown in *E. coli* and other species that AI-2 production and uptake are controlled by catabolite repression through the cyclic AMP (cAMP)-cAMP receptor protein (CRP) complex [7, 37, 38]. In the presence of glucose or other phosphotransferase system (PTS) sugars, low levels of intracellular cAMP and CRP results in reduced transcription of the *lsr* operon. This indirectly causes *luxS* upregulation and enhances AI-2 production and accumulation in the extracellular medium until all available sugars are depleted, after which AI-2 levels fall significantly [7, 38–40]. We assessed the influence of exogenous carbon sources on AI-2 levels and found that the addition of glucose, sucrose and glycerol to LB all significantly increased AI-2 accumulation in the extracellular medium between the mid-exponential and early stationary growth phases. Similar AI-2 detection profiles were observed for the three carbohydrate conditions tested. This was in contrast to Zhu *et al*., who reported distinct AI-2 activity profiles [35]. Specifically, they showed that glucose stimulated *K. pneumoniae* to accumulate up to four-fold more AI-2 than sucrose and glycerol, with peak levels seen at late-exponential growth. In addition, maximum AI-2 levels from glycerol and sucrose supplementation were observed at mid-exponential and early-stationary growth phases, respectively [35]. Genetic variations between the strains used in these studies may account for the differences in AI-2 profiles. For instance, differences in the presence, expression or activity of PTS proteins between bacterial strains to import and utilise certain saccharides could influence catabolite repression systems, and hence QS signalling [38, 41]. Our results, combined with other studies described above, suggest that a relationship between QS and catabolite repression through a cAMP-CRP mechanism may exist in *K. pneumoniae* - further investigation is required to determine this.

### Assessment of biofilm formation by a *K. pneumoniae ΔluxS* mutant

Many bacterial species build surface-attached, multi-cellular communities known as biofilms. These biofilms are associated with increased resistance to antimicrobial agents compared to planktonic cells. Previous research has shown an interplay between quorum sensing and biofilm formation [42, 43]. For instance, AI-2 production or uptake has been shown to influence *E. coli* biofilm formation via an effect on flagellar motion and motility [44], as well as biofilm formation by *Pseudomonas aeruginosa* [45] and *S. enterica* serovar Typhimurium [46]. *K. pneumoniae* forms biofilms on various surfaces, where cell attachment is dependent on the production of exopolysaccharides and adhesive proteins such as type 3 fimbriae [47–50]. There have been limited studies describing a relationship between QS systems and biofilm formation by *K. pneumoniae *. One study demonstrated that although a *K. pneumoniae* LM21 Δ*luxS* mutant did not show major differences in a microtiter plate biofilm assay, the mutant exhibited reduced microcolony development following growth in continuous-flow chambers [5]. In another study, De Araujo *et al*. showed that *K. pneumoniae* LM21 strains deficient in either AI-2 export (Δ*tqsA*) or import (Δ*lsrCD*) machinery demonstrated reduced biofilm thickness but increased surface coverage following growth in a dynamic microfermentor [24]. In addition, *luxS* was shown to be upregulated in biofilm-grown XDR *K. pneumoniae* isolates [51].

In the present study, we imaged *K. pneumoniae* KP563 using SEM and observed evidence of reduced spatial distribution and microcolony formation by the Δ*luxS* mutant. As such, we speculate that AI-2-signalling may have more influence on biofilm architecture than biomass, which was also proposed by others [5, 24]. No major differences in biofilm formation between the wild-type and Δ*luxS* mutant were observed when using a microtiter plate assay, even in carbohydrate-rich media shown to significantly promote AI-2 accumulation. In part, this observation was consistent with the qRT-PCR comparison of wild-type and *ΔluxS* mutant biofilm extracts, which showed no significant differences in the relative expression of *mrkA*, which encodes the major subunit of type 3 fimbriae and known to be an important factor for initial-stage biofilm formation [52, 53]. Therefore, we found no evidence that type 3 fimbriae were regulated by AI-2-mediated QS for the bacterial isolate and assay conditions tested.

We speculate that the reduced biofilm formation of *K. pneumoniae* in the presence of carbohydrate supplementation was the result of enhanced exopolysaccharide synthesis, which could potentially mask the exposure and function of underlying adhesive factors such as fimbriae. Alternatively, a nutrient excess could result in regulatory changes to promote planktonic growth while limiting biofilm growth, the latter of which might instead be favoured in nutrient-poor environments [54]. Carbohydrate supplementation to the growth media is known to significantly increase capsule polysaccharide (*cps*) gene expression and CPS biosynthesis in *K. pneumoniae* [55, 56]. Moreover, CPS biosynthesis by *K. pneumoniae* was shown to be upregulated in response to glucose by cAMP-dependent carbon catabolite repression (CCR) [56]. Therefore, it is possible that a complex regulatory system involving catabolite repression may influence both CPS biosynthesis and AI-2 signalling in *K. pneumoniae *.

Our results are in agreement with other studies showing that biofilm formation by *K. pneumoniae* and *E. coli* could be inhibited by high concentrations of sugars such as glucose [57, 58]. The interference of bacterial capsulation with underlying adhesins to cause reduced bacterial attachment or biofilm formation is a well-recognised observation in *K. pneumoniae *. For instance, capsule expression was shown to block the activity of Antigen 43 [59], and defects in exopolysaccharide synthesis promoted increased bacterial adherence, possibly as a result of more opportunities for cell-surface and/or intercellular interactions [47, 60–63].

### LuxS-mediated gene expression in *K. pneumoniae* biofilms

The polysaccharides comprise a component of the extracellular polymeric substances (EPS) that bacteria within a biofilm are embedded within. The properties of EPS provide stability and architecture to the biofilm, as well as trapping nutrients, preventing desiccation, and preventing antimicrobial access to bacteria [64]. In other bacteria, QS systems have been shown to regulate the expression of polysaccharide production [65–67]. However, in *K. pneumoniae *, the expression of capsule polysaccharide biosynthesis genes (*wza*, *wzi* and *wzx*) were unaltered in *Δlsr*, *Δtqs* and *ΔluxS* mutants during biofilm growth, suggesting that the capsule was not regulated via AI-2 signalling [24].

We observed that the expression of *pgaA*, which encodes the outer-membrane PgaA porin that facilitates PNAG translocation to the cell surface, was upregulated in the *ΔluxS* mutant. PNAG is a common bacterial surface polysaccharide and is an important component of the EPS of biofilms [68–72]. In *K. pneumoniae *, PNAG was shown to be a virulence factor in a murine model of peritonitis and can mediate opsonophagocytosis [73, 74]. Furthermore, *pgaA* was shown to be upregulated in biofilm-grown *K. pneumoniae* [51], and a *K. pneumoniaeΔpgaC* mutant that lacked the β-glycosyltransferase needed to polymerise PNAG showed reduced biofilm formation in the presence of 1 % bile salts [73]. Here, we suggest that *K. pneumoniae* might use AI-2-mediated QS to regulate the expression of PNAG. It is possible that PNAG production is downregulated by bacteria to avoid detection in situations where the extracellular exposure of the antigen would trigger opsonophagocytosis, especially in biofilm communities where the cell density might be high. Another possibility, which is not mutually exclusive depending on the environmental niche, is that AI-2-mediated signalling regulates PNAG expression to alter the opportunities for *K. pneumoniae* to engage in cell-cell and/or cell-surface interactions. We believe this is the first description of a possible relationship between the regulation of PNAG expression and a QS system; however, further experiments are required to characterise the molecular mechanisms involved, including any involvement with the activated methyl cycle.

The LPS is a macromolecule attached to the outer membrane of Gram-negative bacteria and can influence biofilm formation and structure in *K. pneumoniae* and other species through various mechanisms, such as modulating surface hydrophobicity and surface charge [60, 75–78]. The *wbbM* and *wzm* LPS synthesis genes were previously shown to be upregulated in the biofilms of *K. pneumoniae* isolates compared to planktonic growth [51]. Our results showed that *luxS* could have a role in regulating LPS synthesis in the early stages of biofilm formation, because the *wzm* gene, which encodes a membrane protein involved in translocation of the O-antigen, was downregulated in *ΔluxS* mutant 8 h biofilm extracts. Our results are in contrast to the study by De Araujo *et al*., which instead showed upregulation of both the *wbbM* and *wzm* genes in *K. pneumoniae* LM21 *ΔluxS* mutant biofilm extracts compared to wild-type cell extracts [24]. The differences between the two studies could be due to genetic variations in the bacterial isolates and/or the assay conditions used.

In summary, this study established a functional AI-2 QS system in an extensively drug-resistant *K. pneumoniae* clinical isolate and found a relationship between *luxS* and the expression of LPS and PNAG biosynthesis genes, as well as biofilm architecture. Biofilm formation, polysaccharide production and metabolic pathways are influenced by nutrient availability and other environmental conditions. Further investigations of how *K. pneumoniae* coordinates these processes within the QS signalling network, as well as characterising other genes and processes regulated by AI-2, could provide new avenues directed towards reducing the spread and burden of disease caused by this pathogen.
